# Effects of Ocean Warming on the Underexplored Members of the Coral Microbiome

**DOI:** 10.1093/icb/icac005

**Published:** 2022-03-08

**Authors:** Justin Maire, Patrick Buerger, Wing Yan Chan, Pranali Deore, Ashley M Dungan, Matthew R Nitschke, Madeleine J H van Oppen

**Affiliations:** School of BioSciences, University of Melbourne, Parkville, VIC 3010, Australia; School of BioSciences, University of Melbourne, Parkville, VIC 3010, Australia; Applied BioSciences, Macquarie University, Sydney, NSW 2109, Australia; School of BioSciences, University of Melbourne, Parkville, VIC 3010, Australia; School of BioSciences, University of Melbourne, Parkville, VIC 3010, Australia; School of BioSciences, University of Melbourne, Parkville, VIC 3010, Australia; Australian Institute of Marine Science, Townsville, QLD 4810, Australia; School of BioSciences, University of Melbourne, Parkville, VIC 3010, Australia; Australian Institute of Marine Science, Townsville, QLD 4810, Australia

## Abstract

The climate crisis is one of the most significant threats to marine ecosystems. It is leading to severe increases in sea surface temperatures and in the frequency and magnitude of marine heatwaves. These changing conditions are directly impacting coral reef ecosystems, which are among the most biodiverse ecosystems on Earth. Coral-associated symbionts are particularly affected because summer heatwaves cause coral bleaching—the loss of endosymbiotic microalgae (Symbiodiniaceae) from coral tissues, leading to coral starvation and death. Coral-associated Symbiodiniaceae and bacteria have been extensively studied in the context of climate change, especially in terms of community diversity and dynamics. However, data on other microorganisms and their response to climate change are scarce. Here, we review current knowledge on how increasing temperatures affect understudied coral-associated microorganisms such as archaea, fungi, viruses, and protists other than Symbiodiniaceae, as well as microbe-microbe interactions. We show that the coral-microbe symbiosis equilibrium is at risk under current and predicted future climate change and argue that coral reef conservation initiatives should include microbe-focused approaches.

## Introduction

Coral reefs are among the most diverse and productive ecosystems on our planet. Their species richness is largely due to the three-dimensional structure produced by scleractinian corals, and soft corals to a lesser extent, which provides a habitat for more than 25% of all marine multicellular eukaryotic species (Fisher et al., 2015). Their ecological success has been attributed to the symbiotic associations they form with various microorganisms (Blackall et al., 2015; Bourne et al., 2016; Van De Water et al., 2018; van Oppen and Blackall, 2019). Amongst them, intracellular photosynthetic algae of the Symbiodiniaceae family are essential for reef coral survival, as they meet most of their host's energy requirements through translocation of photosynthate (Muscatine, 1967).

**Fig. 1:**
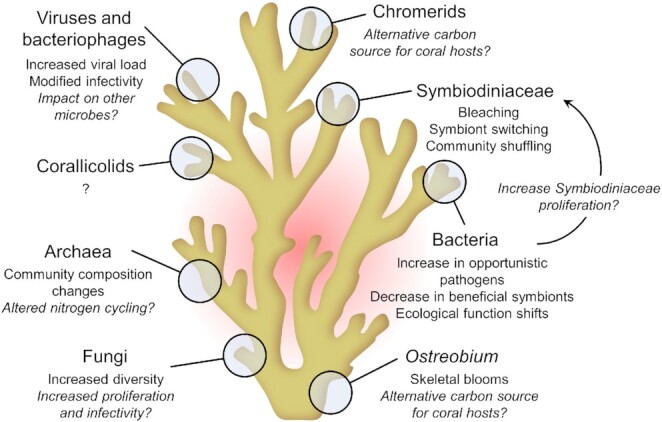
Impact of elevated sea surface temperatures on known coral holobiont members. Under thermal stress, all microorganisms may be affected and in turn impact the health and functioning of the coral host or other microorganisms. Hypotheticals are italicized.

The accumulation of carbon dioxide and other greenhouse gasses, manifested as increased sea surface temperatures and ocean acidification, is severely altering marine environments. These changed conditions drastically impact the coral host animal (Stuart-Smith et al., 2018; Leggat et al., 2019), as well as coral-associated microbial communities (Vanwonterghem and Webster, 2020). The impact of elevated sea surface temperatures on coral-associated Symbiodiniaceae and bacteria has been widely studied, and can lead to shifts in existing microbial communities, acquisition of new microbial partners or even the loss of beneficial symbionts (Fig. 1). One of the most drastic examples of the latter is coral bleaching—the loss of Symbiodiniaceae from coral tissues following prolonged thermal stress (Weis, 2008; Suggett and Smith, 2020), leading to coral starvation, death, and coral reef deterioration (Hughes et al., 2018; Leggat et al., 2019). Altered environmental conditions can also lead to shifts in the specific coral-associated Symbiodiniaceae communities via “shuffling” (i.e., change in the relative abundance of Symbiodiniaceae taxa already present within the host) and/or “switching” (i.e., acquisition of new Symbiodiniaceae taxa from the environment) (Baker, 2003; Boulotte et al., 2016; Claar et al., 2020; Huang et al., 2020; Ros et al., 2021; Scharfenstein et al., 2022). Multiple studies have shown that some corals dominated by the Symbiodiniaceae genus *Cladocopium* become dominated by the more thermally resilient *Durusdinium* from the environment following heat stress events (Berkelmans and van Oppen, 2006; Silverstein et al., 2015; Boulotte et al., 2016). Changes in coral-associated bacterial communities have also been observed in response to thermal stress (Fig. 1) (Ziegler et al., 2017; Grottoli et al., 2018; Savary et al., 2021), although they can remain unaffected (Wessels et al., 2017; Epstein et al., 2019; Ziegler et al., 2019). The factors underlying the flexibility or inflexibility of the microbiome during thermal stress remain unknown, but this trait appears to be coral species-specific. Ziegler et al (2019) hypothesized that coral species with low physiological plasticity would have a more stable microbiome, and vice versa. Thermal stress is often associated with an increase in potential pathogens, such as *Vibrio* (Morrow et al., 2018), and a decrease in potentially beneficial symbionts, such as *Endozoicomonas*, in both scleractinian corals and octocorals (McDevitt-Irwin et al., 2017; Maher et al., 2020; Li et al., 2021; Savary et al., 2021; Tignat-Perrier et al., 2022). Additionally, bacterial virulence or the upregulation of bacterial virulence and secondary metabolism genes are also observed at higher temperatures (Vega Thurber et al., 2009; Littman et al., 2011; Kimes et al., 2012; Garren et al., 2015). While this is a rapidly growing field of research, more functional studies are needed to fully elucidate the impact of bacterial community changes on coral health and coral bleaching.

In addition to Symbiodiniaceae and bacteria, corals associate with a myriad of other microorganisms, such as other protists, archaea, viruses, and fungi, whose roles in the coral holobiont and response to elevated temperatures remain poorly understood (Ainsworth et al., 2017). Here, we review the limited information available on how climate change impacts these underexplored coral–microbe interactions, as well as symbiont–symbiont interactions, and highlight the need to more deeply investigate these members of the coral holobiont.

## Archaea

Coral-associated archaea are often overlooked in metabarcoding and metagenomics studies because read numbers are much lower than their bacterial counterparts (Wegley et al., 2007; Littman et al., 2011), and primers used to target the bacterial 16S rRNA gene often do not detect archaeal 16S rRNA sequences (Eloe-Fadrosh et al., 2016). Nonetheless, archaeal symbionts are known to colonize the coral surface mucus layer (Kellogg, 2004; Frade et al., 2016). Archaeal communities are composed primarily of Euryarchaeota, with the phyla Thaumarchaeota and Crenarchaeota also consistently detected (Kellogg, 2004; Wegley et al., 2004; Littman et al., 2011; Wang et al., 2018). Unlike bacterial communities, the taxonomic affiliation of archaeal communities shows larger overlap with seawater communities and is likely more dependent on geographical location than host taxonomy (Kellogg, 2004; Frade et al., 2016; but see O'Brien et al., 2021). Although archaeal functions are still poorly understood, genomic and metagenomic analyses have pointed at a potential for nitrogen cycling, and specifically for ammonium oxidation in Crenarchaeota (Beman et al., 2007; Siboni et al., 2008, 2012; Robbins et al., 2019). Archaeal genes involved in carbon fixation and vitamin B_12_ biosynthesis have also been detected in several coral species (Kimes et al., 2010; Robbins et al., 2019), but whether they translocate any of this carbon to the coral host is unknown. Study of carbon translocation genes and isotope labeling of cultured archaea followed by reinoculation in corals may shed light on this aspect.

Few studies have assessed the impact of thermal stress on coral-associated archaeal communities (Fig. 1). The overall relative abundance of archaea was found to decrease in bleached *Acropora millepora* (Littman et al., 2011). Additionally, thermal stress was linked with a decrease in the relative abundance of Euryarchaeota and an increase in Crenarchaeota in both *A. millepora* and *Porites compressa* (Vega Thurber et al., 2009; Littman et al., 2011). The hypothetical role of Crenarchaeota in ammonium oxidation means they may compete for ammonium with Symbiodiniaceae, which are nitrogen-limited *in hospite* and prefer ammonium over nitrate as a nitrogen source (Rädecker et al., 2015). Additionally, environmental mesophilic archaea were shown to exhibit increased metabolic activity under increased temperatures (Smith et al., 2019), which may increase ammonium oxidation rates. An increase in ammonia oxidation by the archaeal community may counteract the ammonia increase from host catabolism observed during early thermal stress (Baker et al., 2018; Rädecker et al., 2021b). This may assist in maintaining Symbiodiniaceae's nitrogen-limited state and control of their proliferation rate and the amount of photosynthate that is transferred to the host. Archaeal abundance in the coral holobiont is relatively low, and their ammonia oxidation rates have not yet been investigated. It is therefore possible that their involvement in nitrogen cycling is negligible compared to the other members of the holobiont. For example, diazotrophs may compensate an increase in nitrogen removal by archaea, as their nitrogen-fixing activity can increase during heat stress (Santos et al., 2014; Cardini et al., 2016; Rädecker et al., 2021a). Hence, the degree to which archaea may impact holobiont nitrogen cycling and in turn Symbiodiniaceae functioning should be investigated further.

## Fungi

Corals harbor diverse fungal communities, dominated by Ascomycetes and Basidiomycetes, most of which are thought to be endolithic (Bentis et al., 2000; Gleason et al., 2017; Góes-Neto et al., 2020). Octocorals also harbor complex fungal communities, although culture-independent data are lacking (Van De Water et al., 2018). Endolithic communities actively penetrate the coral carbonate skeleton by chemical means and are major players in tropical reef bioerosion (Tribollet, 2008). Most emphasis has been placed on fungi as potential pathogens and skeletal borers. For example, *Aspergillus sydowii* is a pathogen of sea fans (Harvell et al., 1999), and corals lay down dense skeletal layers when endolithic fungal hyphae approach living coral tissue (Le Campion-Alsumard et al., 1995; Bentis et al., 2000). Endolithic fungi also play important roles in the global calcium carbonate cycle by participating in the bioerosion of coral skeleton (Gleason et al., 2017). Metagenomic analyses suggest they may also be involved in nitrogen and carbon cycling within coral holobionts (Wegley et al., 2007; Kimes et al., 2010).

Coral-associated fungal communities can be affected by increases in sea surface temperatures (Fig. 1). Phylogenetic diversity was higher in fungal communities associated with *Acropora hyacinthus* sampled from warm reef pools, compared with conspecific colonies from naturally colder, adjacent pools (Amend et al., 2011). Further, total fungal DNA was found to increase in thermally stressed *P. compressa* (Vega Thurber et al., 2009), suggesting an increase in abundance of fungi with elevated temperature. In plant–mycorrhizal associations, elevated temperatures often result in increased fungal extra-radical hyphae size, thereby expanding the surface for fungus–soil exchanges (Bennett and Classen, 2020; Chanda et al., 2020). This may impact fungal metabolic capabilities and lead to improved respiration and carbon distribution in the case of arbuscular mycorrhizal fungi (Chanda et al., 2020). Future investigations should focus on assessing changes in coral-associated fungal community composition and functional roles under thermal stress.

## Ostreobium

Along with fungi, coral endolithic communities include filamentous algae (Chlorophytes and Rhodophytes) (Tribollet, 2008; Marcelino and Verbruggen, 2016), and sometimes green sulfur bacteria (Yang et al., 2019). The photosynthetic *Ostreobium* spp. (Chlorophytes) (Iha et al., 2021) often dominate coral endolithic communities, and can form visible green bands in adult coral skeletons (Ricci et al., 2019; Pernice et al., 2020). Primary polyps (7-day-old *Pocillopora damicornis*) can already be colonized by *Ostreobium* (Massé et al., 2018). The recently sequenced genome of *Ostreobium* reveals unique adaptations to the darker and extremely variable endolithic environment. This includes a rich repertoire of light-harvesting complex proteins and genes involved in the oxidative stress response, as well as the absence of many genes involved in photoprotection and photoreception (Iha et al., 2021). Additionally, *Ostreobium* can translocate photosynthate to coral tissues (Sangsawang et al., 2017).

After coral bleaching, the absence of Symbiodiniaceae in coral tissues results in increased light availability to the skeleton (Enríquez et al., 2005) and *Ostreobium* blooms (Diaz-Pulido and McCook, 2002; Fine et al., 2006; Galindo-Martínez et al., 2022), which may have potential benefits to other members of the coral holobiont (Fig. 1). For example, thermally bleached *Orbicella falveolata* colonies with *Ostreobium* blooms were able to recover, while colonies without *Ostreobium* blooms did not (Galindo-Martínez et al., 2022). First, this increased abundance of *Ostreobium* in the coral skeleton may reduce skeletal light scattering and limit light stress for the remaining Symbiodiniaceae (Enríquez et al., 2005; Yamazaki et al., 2008; Galindo-Martínez et al., 2022). Second, photosynthate translocated by *Ostreobium* may provide an alternate source of energy for bleached corals, thereby partly compensating the loss of photosynthate translocation from Symbiodiniaceae (Fine and Loya, 2002; Sangsawang et al., 2017). In *Oculina patagonica, Ostreobium* showed increased carbon translocation rates in bleached corals, compared to healthy corals (Fine and Loya, 2002), and may thereby limit starvation and promote coral recovery.

## Other protists

Many other protists beside Symbiodiniaceae and *Ostreobium* are known to associate with corals, including alveolates (Clerissi et al., 2018), such as chromerids and corallicoids. Corallicolids were formally described only recently as part of the mostly parasitic taxon Apicomplexa (Kwong et al., 2019, 2021), although they had been detected in previous amplicon studies (Toller et al., 2002; Clerissi et al., 2018). They are located intracellularly within the coral's mesenterial filaments (Kwong et al., 2019), although whether they are mutualistic or parasitic remains unknown. Corallicolids have maintained some of the cellular machinery to synthesize chlorophyll, although their plastid genome lacks photosystem genes, suggesting that they are unlikely able to photosynthesize and that their plastid could be an apicoplast—a vestigial, non-photosynthetic plastid (Kwong et al., 2019). Corallicolids were detected in corals at depths as great as 1400 m (Vohsen et al., 2020), suggesting they might be mixotrophic or non-photosynthetic at all. However, the function of chlorophyll synthesis related genes in corallicolids remains unknown. Having been discovered very recently, the effects of environmental stressors, including high temperatures, on corallicolids and coral–corallicolid interactions have not yet been examined.

Chromerids were first described more than 10 years ago after being isolated from the temperate coral *Plesiastrea versipora* (Moore et al., 2008), and can colonize various other tropical corals (Cumbo et al., 2013). Chromerids possess a chloroplast and are capable of photosynthesis (Moore et al., 2008; Oborník et al., 2012; Chakravarti et al., 2019), and may therefore be beneficial for corals, in a similar way to Symbiodiniaceae. One study has assessed the effect of elevated temperatures on the health and performance of chromerids, both in culture and *in hospite* (Fig. 1) (Chakravarti et al., 2019). Cultured chromerids of two species showed higher photochemical health above 30°C, when compared with the Symbiodiniaceae *Cladocopium* C1^acro^ (formerly known as *Cladocopium goreaui* (Beltrán et al., 2021)). While uptake of chromerids by larvae of two *Acropora* species was minimal compared to *Cladocopium* C1^acro^, larvae colonized by *Chromera velia* exhibited higher survival rates to thermal stress (Chakravarti et al., 2019). We hypothesize that, like *Ostreobium* algae, chromerids may act as alternative carbon sources in thermally stressed and bleached corals, although their typically low *in hospite* density may limit their nutritional impact.

## Viruses

Viruses are present in relatively large abundances in the water column, outnumbering bacteria by an order of magnitude (Bergh et al., 1989). They have a high diversity in hard and soft corals (Weynberg et al., 2015; Gudenkauf and Hewson, 2016) and can target every member of the holobiont as a potential host (Vega Thurber et al., 2017), i.e., eukaryotic viruses interact with corals and eukaryotic microbes as hosts, while bacteriophages and archaeal viruses infect bacteria and archaea, respectively. Viruses are believed to have versatile roles during their interactions with the different members of the coral holobiont and can be drivers of disease (Buerger and van Oppen, 2018). For example, lysogenic bacteriophages that integrate into the genome of infected bacteria may increase bacterial virulence and alter their functionality, as suggested for the virulence of *Vibrio coralliilyticus* related to the coral disease white syndrome (Weynberg et al., 2015). Other coral diseases have been directly linked to viral activities, such as yellow band/blotch disease (Cervino et al., 2004) and white plague disease (Soffer et al., 2013). However, bacteriophages can also prevent diseases and control bacterial populations(Buerger et al., 2016). For example, when bacteriophages infect some pathogenic bacteria and take over the cell machinery for propagation, lysis of the infected bacteria may reduce the impact of a bacterial disease (Bull and Gill, 2014; Vega Thurber et al., 2017). As such, bacteriophages used in phage therapy have successfully prevented white plague disease in *Favia favus* when administered at the same time as the pathogen and limited coral-coral transmission (Efrony et al., 2009).

Increased seawater temperature can affect the activity and dynamics of these host–virus interactions (Fig. 1) (Vega Thurber et al., 2017). Several studies have shown an increase in viral reads during thermal stress (Marhaver et al., 2008; Vega Thurber et al., 2009; Nguyen-Kim et al., 2015; Correa et al., 2016; Messyasz et al., 2020) Coral heat resilience may also be affected by viral activity, since heat stress in corals can trigger an increase in activity or abundance of DNA- and RNA-viruses that infect Symbiodiniaceae (Levin et al., 2016; Grupstra et al., 2021), which may result in increased Symbiodiniaceae lysis. However, while increased seawater temperatures may provide more optimal conditions for some virus-host dynamics and an increase in viral production, for others it may result in reduced optimal conditions for virus propagation and activity (Danovaro et al., 2011). It is therefore difficult to predict a directional shift for the overall coral–virus interactions and dynamics.

## Symbiodiniaceae–bacteria interactions

While coral interactions with Symbiodiniaceae and bacteria are often at the forefront of research, Symbiodiniaceae–bacteria associations remain largely understudied (Matthews et al., 2020). Indeed, cultured Symbiodiniaceae have been shown to associate with diverse communities of bacteria (Frommlet et al., 2015; Lawson et al., 2018; Nitschke et al., 2020; Maire et al., 2021b), and intracellular bacteria were observed in cultured and *in hospite* Symbiodiniaceae (Maire et al., 2021b). While the functions of such bacterial symbionts remain elusive, co-cultivation of bacterial consortia with microalgae other than Symbiodiniaceae improved microalgal carbon conversion efficiency and significantly enhanced biomass yields (Vasseur et al., 2012; Bell et al., 2016). The growth enhancing role of symbiotic bacteria is mainly attributed to the release of growth promoting factors such as indole acetic acid (De-Bashan et al., 2008) and vitamin B_12_ (Croft et al., 2005). As Symbiodiniaceae are also auxotrophic for vitamin B_12_ (Agostini et al., 2009), it is expected that the metabolic associations between Symbiodiniaceae and symbiotic bacteria partly resemble other microalgal systems. Co-cultivation of *Chlamydomonas reinhardtii* with vitamin B_12_-producing bacteria enhanced its thermal tolerance (Xie et al., 2013), suggesting live bacteria and bacterial metabolites might confer thermal tolerance to microalgae, including Symbiodiniaceae. In line with this, addition of an algal phycosphere-associated bacterium belonging to the *Muricauda* genus to an antibiotic-treated Symbiodiniaceae culture (*Durusdinium* sp.) was recently shown to restore the algal heat tolerance (Motone et al., 2020)). It is important to understand how Symbiodiniaceae-bacteria interactions may influence Symbiodiniaceae and coral holobiont thermal tolerance, and how increased temperatures will impact Symbiodiniaceae-associated bacteria. To date, only one study monitored Symbiodiniaceae-associated bacteria during a heat stress experiment. Increased temperatures resulted in increased bacterial numbers and changes in the bacterial community composition in cultured Symbiodiniaceae (Camp et al., 2020). Bacterial communities were more stable in association with *Durusdinium trenchii*, which is more thermally tolerant than the other cultures from this study (Camp et al., 2020). Whether bacterial community stability is a cause or a consequence of enhanced thermal tolerance remains unknown (Camp et al., 2020).

## Conclusions

Elevated sea surface temperatures are a major threat to marine ecosystems, including coral reefs. Not only does climate change drive coral bleaching (the loss of Symbiodiniaceae), but temperature anomalies also affect other coral-associated microorganisms with changes in microorganism community composition and function likely impacting coral health and thermal resistance. While these symbiotic imbalances are clear in the case of Symbiodiniaceae, additional work is needed to understand the extent of thermal stress on other coral-associated microbes (Ainsworth et al., 2017).

The relatively low number of studies for these underexplored microbes is partly due to technical challenges, such as their low abundance, and the inability to specifically sequence their genomic material (e.g., 18S rRNA coral or Symbiodiniaceae sequences would far outweigh similar sequences from other micro-eukaryotes, making the latter harder to characterize). However, techniques such as fluorescence-activated cell-sorting, or laser capture-microdissection for the bigger symbionts, can be used to stain and sort different cell populations and enrich targeted symbionts (Rosental et al., 2017; Maire et al., 2021a). These populations can subsequently be used for meta-omics experiments, while minimizing host or Symbiodiniaceae contamination. Spatial approaches, such as metabolite distribution mapping with matrix-assisted laser desorption/ionization (MALDI-MSI), or spatial genomics and proteomics through NanoString technologies may also assist in obtaining functional information on specific symbionts. Higher resolution techniques, such as nanoscale secondary ion mass spectrometry (NanoSIMS) or elemental analysis coupled with electron microscopy, may be needed for smaller symbionts like viruses and archaea. As it is also very challenging to culture most of these symbionts, functional data rely on culture-independent techniques such as meta-omics and advanced visualization studies. Genome data can inform on the requirements for culturing, thereby providing a potential path to *in vitro* functional characterization.

Microbiome manipulation has been proposed as a tool to enhance certain coral traits, such as thermal bleaching tolerance. Bacterial microbiome transplantation and probiotics (Doering et al., 2021; Santoro et al., 2021) and the introduction of experimentally evolved Symbiodiniaceae into coral (Chakravarti et al., 2017; Buerger et al., 2020) are showing promising results in enhancing coral thermal resistance. While most of the proposed approaches currently focus on Symbiodiniaceae and bacteria (van Oppen and Blackall, 2019; Chan et al., 2021; Peixoto et al., 2021; Maire and van Oppen, 2022), the symbionts and interactions highlighted in this review may represent a yet untapped resource for microbial-mediated conservation approaches. Some methods, like phage therapy, have already been trialed and show promising results (Efrony et al., 2009). However, the lack of fundamental data on these understudied symbionts (e.g., whether corallicolids and chromerids are mutualistic or parasitic) is hampering the development of such approaches. Understanding which symbionts are mutualistic and may provide benefits, and how their interactions with other coral holobiont members are affected by thermal stress will be key in expanding our microbial arsenal for coral bleaching mitigation.

## Data Availability

No new data were generated or analyzed in support of this research.

## References

[bib1] Agostini S , Suzuki Y, Casareto BE, Nakano Y, Hidaka M, Badrun N. 2009. Coral symbiotic complex: Hypothesis through vitamin B12 for a new evaluation. Galaxea, J Coral Reef Stud. 11:1–11.

[bib2] Ainsworth TD , Fordyce AJ, Camp EF. 2017. The Other Microeukaryotes of the Coral Reef Microbiome. Trends Microbiol. 25:980–91. 28720387 10.1016/j.tim.2017.06.007

[bib3] Amend AS , Barshis DJ, Oliver TA. 2012. Coral-associated marine fungi form novel lineages and heterogeneous assemblages. ISME J. 6:1291–301. 22189500 10.1038/ismej.2011.193 PMC3379630

[bib4] Baker AC. 2003. Flexibility and Specificity in Coral-Algal Symbiosis: Diversity, Ecology, and Biogeography of Symbiodinium. Annu Rev Ecol Evol Syst. 34:661–89.

[bib5] Baker DM , Freeman CJ, Wong JCY, Fogel ML, Knowlton N. 2018. Climate change promotes parasitism in a coral symbiosis. ISME J. 12:921–30. 29379177 10.1038/s41396-018-0046-8 PMC5864192

[bib6] Bell TAS , Prithiviraj B, Wahlen BD, Fields MW, Peyton BM. 2016. A Lipid-Accumulating Alga Maintains Growth in Outdoor Alkaliphilic Raceway Pond with Mixed Microbial Communities. Front Microbiol. 6:1480. 26779138 10.3389/fmicb.2015.01480 PMC4703792

[bib7] Beltrán VH , Puill-Stephan E, Howells E, Flores-Moya A, Doblin M, Núñez-Lara E, Escamilla V, López T, van Oppen MJH. 2021. Physiological diversity among sympatric, conspecific endosymbionts of coral (Cladocopium C1acro) from the Great Barrier Reef. Coral Reefs. 40:985–97.

[bib8] Beman JM , Roberts KJ, Wegley L, Rohwer F, Francis CA. 2007. Distribution and Diversity of Archaeal Ammonia Monooxygenase Genes Associated with Corals. Appl Environ Microbiol. 73:5642. 17586663 10.1128/AEM.00461-07 PMC2042080

[bib9] Bennett AE , Classen AT. 2020. Climate change influences mycorrhizal fungal–plant interactions, but conclusions are limited by geographical study bias. Ecology. 101:e02978. 31953955 10.1002/ecy.2978

[bib10] Bentis CJ , Kaufman L, Golubic S. 2000. Endolithic fungi in reef-building corals (Order : Scleractinia) are common, cosmopolitan, and potentially pathogenic. Biol Bull. 198:254–60. 10786945 10.2307/1542528

[bib11] Bergh Ø , BØrsheim KY, Bratbak G, Heldal M. 1989. High abundance of viruses found in aquatic environments. Nature. 340:467–8. 2755508 10.1038/340467a0

[bib12] Berkelmans R , van Oppen MJH. 2006. The role of zooxanthellae in the thermal tolerance of corals: a “nugget of hope” for coral reefs in an era of climate change. Proceedings Biol Sci. 273:2305–12. 10.1098/rspb.2006.3567 PMC1636081 16928632

[bib13] Blackall LL , Wilson B, van Oppen MJH. 2015. Coral-the world's most diverse symbiotic ecosystem. Mol Ecol. 24:5330–47. 26414414 10.1111/mec.13400

[bib14] Boulotte NM , Dalton SJ, Carroll AG, Harrison PL, Putnam HM, Peplow LM, van Oppen MJH. 2016. Exploring the Symbiodinium rare biosphere provides evidence for symbiont switching in reef-building corals. ISME J. 10:2693. 27093048 10.1038/ismej.2016.54 PMC5113844

[bib15] Bourne DG , Morrow KM, Webster NS. 2016. Insights into the coral microbiome: Underpinning the health and resilience of reef ecosystems. Annu Rev Microbiol. 70:317–40. 27482741 10.1146/annurev-micro-102215-095440

[bib16] Buerger P , Alvarez-Roa C, Coppin CW, Pearce SL, Chakravarti LJ, Oakeshott JG, Edwards OR, van Oppen MJH. 2020. Heat-evolved microalgal symbionts increase coral bleaching tolerance. Sci Adv. 6:eaba2498. 32426508 10.1126/sciadv.aba2498 PMC7220355

[bib17] Buerger P , van Oppen MJ. 2018. Viruses in corals: hidden drivers of coral bleaching and disease?. Microbiol Aust. 39:9.

[bib18] Buerger P , Wood-Charlson EM, Weynberg KD, Willis BL, van Oppen MJH. 2016. CRISPR-Cas defense system and potential prophages in cyanobacteria associated with the coral black band disease. Front Microbiol. 7:2077. 28066391 10.3389/fmicb.2016.02077 PMC5177637

[bib19] Bull JJ , Gill JJ. 2014. The habits of highly effective phages: Population dynamics as a framework for identifying therapeutic phages. Front Microbiol. 5:618. 25477869 10.3389/fmicb.2014.00618 PMC4235362

[bib20] Camp EF , Kahlke T, Nitschke MR, Varkey D, Fisher NL, Fujise L, Goyen S, Hughes DJ, Lawson CA, Ros M, et al. 2020. Revealing changes in the microbiome of Symbiodiniaceae under thermal stress. Environ Microbiol. 22:1294–309. 31997503 10.1111/1462-2920.14935

[bib21] Cardini U , van Hoytema N, Bednarz VN, Rix L, Foster RA, Al-Rshaidat MMD, Wild C. 2016. Microbial dinitrogen fixation in coral holobionts exposed to thermal stress and bleaching. Environ Microbiol. 18:2620–33. 27234003 10.1111/1462-2920.13385

[bib22] Cervino JM , Hayes R, Goreau TJ, Smith GW. 2004. Zooxanthellae Regulation in Yellow Blotch/Band and Other Coral Diseases Contrasted with Temperature Related Bleaching: In Situ Destruction vs Expulsion. Symbiosis. 37:63–85.

[bib23] Chakravarti LJ , Beltran VH, van Oppen MJH. 2017. Rapid thermal adaptation in photosymbionts of reef-building corals. Global Change Biol. 23:4675–88. 10.1111/gcb.13702 28447372

[bib24] Chakravarti LJ , Negri AP, van Oppen MJH. 2019. Thermal and herbicide tolerances of Chromerid algae and their ability to form a symbiosis with corals. Front Microbiol. 10:173. 30809207 10.3389/fmicb.2019.00173 PMC6379472

[bib25] Chan WY , Oakeshott JG, Buerger P, Edwards OR, van Oppen MJH. 2021. Adaptive responses of free-living and symbiotic microalgae to simulated future ocean conditions. Global Change Biol. 27:1737–54. 10.1111/gcb.15546 33547698

[bib26] Chanda A , Maghrawy H, Sayour H, Gummadidala PM, Gomaa OM. 2020. Impact of Climate Change on Plant-Associated Fungi. In: Ewis Omran E, Negm Aeditors. Climate Change Impacts on Agriculture and Food Security in Egypt. Cham: Springer. p. 83–96.

[bib27] Claar DC , Starko S, Tietjen KL, Epstein HE, Cunning R, Cobb KM, Baker AC, Gates RD, Baum JK. 2020. Dynamic symbioses reveal pathways to coral survival through prolonged heatwaves. Nat Commun. 11:1–10. 33293528 10.1038/s41467-020-19169-y PMC7723047

[bib28] Clerissi C , Brunet S, Vidal-Dupiol J, Adjeroud M, Lepage P, Guillou L, Escoubas J-M, Toulza E. 2018. Protists within corals: The hidden diversity. Front Microbiol. 9:2043. 30233528 10.3389/fmicb.2018.02043 PMC6127297

[bib29] Correa AMS , Ainsworth TD, Rosales SM, Thurber AR, Butler CR, Vega Thurber RL. 2016. Viral Outbreak in Corals Associated with an In Situ Bleaching Event: Atypical Herpes-Like Viruses and a New Megavirus Infecting Symbiodinium. Front Microbiol. 7:127. 26941712 10.3389/fmicb.2016.00127 PMC4761846

[bib30] Croft MT , Lawrence AD, Raux-Deery E, Warren MJ, Smith AG. 2005. Algae acquire vitamin B12 through a symbiotic relationship with bacteria. Nature. 438:90–3. 16267554 10.1038/nature04056

[bib31] Cumbo VR , Baird AH, Moore RB, Negri AP, Neilan BA, Salih A, van Oppen MJH, Wang Y, Marquisc CP. 2013. Chromera velia is Endosymbiotic in Larvae of the Reef Corals Acropora digitifera and A. tenuis. Protist. 164:237–44. 23063731 10.1016/j.protis.2012.08.003

[bib32] Danovaro R , Corinaldesi C, Dell'Anno A, Fuhrman JA, Middelburg JJ, Noble RT, Suttle CA. 2011. Marine viruses and global climate change. FEMS Microbiol Rev. 35:993–1034. 21204862 10.1111/j.1574-6976.2010.00258.x

[bib33] De-Bashan LE , Antoun H, Bashan Y. 2008. Involvement of indole-3-acetic acid produced by the growth-promoting bacterium Azospirillum spp. on promoting growth of Chlorella vulgaris. J Phycol. 44:938–47. 27041612 10.1111/j.1529-8817.2008.00533.x

[bib34] Diaz-Pulido G , McCook LJ. 2002. The fate of bleached corals: patterns and dynamics of algal recruitment. Mar Ecol Prog Ser. 232:115–28.

[bib35] Doering T , Wall M, Putchim L, Rattanawongwan T, Schroeder R, Hentschel U, Roik A. 2021. Towards enhancing coral heat tolerance: a “microbiome transplantation” treatment using inoculations of homogenized coral tissues. Microbiome. 9:102. 33957989 10.1186/s40168-021-01053-6 PMC8103578

[bib36] Efrony R , Atad I, Rosenberg E. 2009. Phage therapy of coral white plague disease: Properties of phage BA3. Curr Microbiol. 58:139–45. 18923867 10.1007/s00284-008-9290-x

[bib37] Eloe-Fadrosh EA , Ivanova NN, Woyke T, Kyrpides NC. 2016. Metagenomics uncovers gaps in amplicon-based detection of microbial diversity. Nat Microbiol. 1:15032. 27572438 10.1038/nmicrobiol.2015.32

[bib38] Enríquez S , Méndez ER, Iglesias-Prieto R. 2005. Multiple scattering on coral skeletons enhances light absorption by symbiotic algae. Limnol Oceanogr. 50:1025–32.

[bib39] Epstein HE , Torda G, van Oppen MJH. 2019. Relative stability of the Pocillopora acuta microbiome throughout a thermal stress event. Coral Reefs. 38:373.

[bib40] Fine M , Loya Y. 2002. Endolithic algae: An alternative source of photoassimilates during coral bleaching. Proc R Soc Lond B Biol Sci. 269:1205–10. 10.1098/rspb.2002.1983 PMC1691023 12065035

[bib41] Fine M , Roff G, Ainsworth TD, Hoegh-Guldberg O. 2006. Phototrophic microendoliths bloom during coral “white syndrome.”. Coral Reefs. 25:577–81.

[bib42] Fisher R , O'Leary RA, Low-Choy S, Mengersen K, Knowlton N, Brainard RE, Caley MJ. 2015. Species richness on coral reefs and the pursuit of convergent global estimates. Curr Biol. 25:500–5. 25639239 10.1016/j.cub.2014.12.022

[bib43] Frade PR , Roll K, Bergauer K, Herndl GJ. 2016. Archaeal and Bacterial Communities Associated with the Surface Mucus of Caribbean Corals Differ in Their Degree of Host Specificity and Community Turnover Over Reefs. PLoS One. 11:e0144702. 26788724 10.1371/journal.pone.0144702 PMC4720286

[bib44] Frommlet JC , Sousa ML, Alves A, Vieira SI, Suggett DJ, Serôdio J. 2015. Coral symbiotic algae calcify ex hospite in partnership with bacteria. Proc Natl Acad Sci USA. 112:6158–63. 25918367 10.1073/pnas.1420991112 PMC4434719

[bib45] Galindo-Martínez CT , Weber M, Avila-Magaña V, Enríquez S, Kitano H, Medina M, Iglesias-Prieto R. 2022. The role of the endolithic alga Ostreobium spp. during coral bleaching recovery. Sci Rep. 12:1–12. 35194106 10.1038/s41598-022-07017-6 PMC8863988

[bib46] Garren M , Son K, Tout J, Seymour JR, Stocker R. 2015. Temperature-induced behavioral switches in a bacterial coral pathogen. ISME JISME J. 10:1363–72. 10.1038/ismej.2015.216 PMC5029190 26636553

[bib47] Gleason FH , Gadd GM, Pitt JI, Larkum AWD. 2017. The roles of endolithic fungi in bioerosion and disease in marine ecosystems. I. General concepts. Mycology. 8:205. 30123641 10.1080/21501203.2017.1352049 PMC6059151

[bib48] Góes-Neto A , Marcelino VR, Verbruggen H, da Silva FF, Badotti F. 2020. Biodiversity of endolithic fungi in coral skeletons and other reef substrates revealed with 18S rDNA metabarcoding. Coral Reefs. 39:229–38.

[bib49] Grottoli AG , Dalcin Martins P, Wilkins MJ, Johnston MD, Warner ME, Cai W-J, Melman TF, Hoadley KD, Pettay DT, Levas S, et al. 2018. Coral physiology and microbiome dynamics under combined warming and ocean acidification. PLoS One. 13:e0191156. 29338021 10.1371/journal.pone.0191156 PMC5770069

[bib50] Grupstra CG , Howe-Kerr LI, Veglia AJ, Bryant RL, Coy SR, Blackwelder PL, Correa AMS. 2021. Thermal stress triggers productive viral infection of a key coral reef symbiont. bioRxiv, 2021.03.17.435810. 10.1038/s41396-022-01194-y PMC9038915 35046559

[bib51] Gudenkauf BM , Hewson I. 2016. Comparative metagenomics of viral assemblages inhabiting four phyla of marine invertebrates. Front Mar Sci. 3:23.

[bib52] Harvell CD , Kim K, Burkholder JM, Colwell RR, Epstein PR, Grimes DJ, Hofmann EE, Lipp EK, Osterhaus AD, Overstreet RM, et al. 1999. Emerging marine diseases—climate links and anthropogenic factors. Science. 285:1505–10. 10498537 10.1126/science.285.5433.1505

[bib53] Huang Y-Y , Carballo-Bolaños R, Kuo C-Y, Keshavmurthy S, Chen CA. 2020. Leptoria phrygia in Southern Taiwan shuffles and switches symbionts to resist thermal-induced bleaching. Sci Rep. 10:7808. 32385394 10.1038/s41598-020-64749-z PMC7210888

[bib54] Hughes TP , Anderson KD, Connolly SR, Heron SF, Kerry JT, Lough JM, Baird AH, Baum JK, Berumen ML, Bridge TC, et al. 2018. Spatial and temporal patterns of mass bleaching of corals in the Anthropocene. Science. 359:80–3. 29302011 10.1126/science.aan8048

[bib55] Iha C , Dougan KE, Varela JA, Avila V, Jackson CJ, Bogaert KA, Chen Y, Judd LM, Wick R, Holt KE, et al. 2021. Genomic adaptations to an endolithic lifestyle in the coral-associated alga Ostreobium. Curr Biol. 31:1393–1402.e5. 33548192 10.1016/j.cub.2021.01.018

[bib56] Kellogg CA. 2004. Tropical Archaea: diversity associated with the surface microlayer of corals. Mar Ecol Prog Ser. 273:81–8.

[bib57] Kimes NE , Grim CJ, Johnson WR, Hasan NA, Tall BD, Kothary MH, Kiss H, Munk AC, Tapia R, Green L, et al. 2012. Temperature regulation of virulence factors in the pathogen Vibrio coralliilyticus. ISME J. 6:835. 22158392 10.1038/ismej.2011.154 PMC3309362

[bib58] Kimes NE , Van Nostrand JD, Weil E, Zhou J, Morris PJ. 2010. Microbial functional structure of Montastraea faveolata, an important Caribbean reef-building coral, differs between healthy and yellow-band diseased colonies. Environ Microbiol. 12:541–56. 19958382 10.1111/j.1462-2920.2009.02113.x

[bib59] Kwong WK , del Campo J, Mathur V, Vermeij MJA, Keeling PJ. 2019. A widespread coral-infecting apicomplexan with chlorophyll biosynthesis genes. Nature. 568:103–7. 30944491 10.1038/s41586-019-1072-z

[bib60] Kwong WK , Irwin NAT, Mathur V, Na I, Okamoto N, Vermeij MJA, Keeling PJ. 2021. Taxonomy of the Apicomplexan Symbionts of Coral, including Corallicolida ord. nov., Reassignment of the Genus Gemmocystis, and Description of New Species Corallicola aquarius gen. nov. sp. nov. and Anthozoaphila gnarlus gen. nov. sp. nov. J Eukaryot Microbiol. 68:e12852. 10.1111/jeu.12852 33768669

[bib61] Lawson CA , Raina J-B, Kahlke T, Seymour JR, Suggett DJ. 2018. Defining the core microbiome of the symbiotic dinoflagellate, *Symbiodinium*. Environ Microbiol Rep. 10:7–11. 29124895 10.1111/1758-2229.12599

[bib62] Le Campion-Alsumard T , Golubic S, Priess K. 1995. Fungi in corals: symbiosis or disease? Interaction between polyps and fungi causes pearl-like skeleton biomineralization on JSTOR. Mar Ecol Prog Ser. 117:137–47.

[bib63] Leggat WP , Camp EF, Suggett DJ, Heron SF, Fordyce AJ, Gardner S, Deakin L, Turner M, Beeching LJ, Kuzhiumparambil U, et al. 2019. Rapid coral decay is associated with marine heatwave mortality events on reefs. Curr Biol. 29:2723. 31402301 10.1016/j.cub.2019.06.077

[bib64] Levin RA , Voolstra CR, Weynberg KD, Van Oppen MJH. 2016. Evidence for a role of viruses in the thermal sensitivity of coral photosymbionts. ISME J. 11:808–12. 27911439 10.1038/ismej.2016.154 PMC5322306

[bib65] Li J , Long L, Zou Y, Zhang S. 2021. Microbial community and transcriptional responses to increased temperatures in coral Pocillopora damicornis holobiont. Environ Microbiol. 23:826–43. 32686311 10.1111/1462-2920.15168 PMC7984454

[bib66] Littman R , Willis BL, Bourne DG. 2011. Metagenomic analysis of the coral holobiont during a natural bleaching event on the Great Barrier Reef. Environ Microbiol Rep. 3:651–60. 23761353 10.1111/j.1758-2229.2010.00234.x

[bib67] Maher RL , Schmeltzer ER, Meiling S, McMinds R, Ezzat L, Shantz AA, Adam TC, Schmitt RJ, Holbrook SJ, Burkepile DE, et al. 2020. Coral Microbiomes Demonstrate Flexibility and Resilience Through a Reduction in Community Diversity Following a Thermal Stress Event. Front Ecol Evol. 8:356.

[bib68] Maire J , Blackall LL, van Oppen MJH. 2021a. Intracellular Bacterial Symbionts in Corals: Challenges and Future Directions. Microorganisms. 9:2209. 34835335 10.3390/microorganisms9112209 PMC8619543

[bib69] Maire J , Girvan SK, Barkla SE, Perez-Gonzalez A, Suggett DJ, Blackall LL, van Oppen MJH. 2021b. Intracellular bacteria are common and taxonomically diverse in cultured and in hospite algal endosymbionts of coral reefs. ISME J. 15:2028–42. 33558689 10.1038/s41396-021-00902-4 PMC8245515

[bib70] Maire J , van Oppen MJH. 2022. A role for bacterial experimental evolution in coral bleaching mitigation?. Trends Microbiol. 30:217–28. 34429226 10.1016/j.tim.2021.07.006

[bib71] Marcelino VR , Verbruggen H. 2016. Multi-marker metabarcoding of coral skeletons reveals a rich microbiome and diverse evolutionary origins of endolithic algae. Sci Rep. 6:31508. 27545322 10.1038/srep31508 PMC4992875

[bib72] Marhaver KL , Edwards RA, Rohwer F. 2008. Viral communities associated with healthy and bleaching corals. Environ Microbiol. 10:2277–86. 18479440 10.1111/j.1462-2920.2008.01652.x PMC2702503

[bib73] Massé A , Domart-Coulon I, Golubic S, Duché D, Tribollet A. 2018. Early skeletal colonization of the coral holobiont by the microboring Ulvophyceae Ostreobium sp. Sci Rep. 8:1–11. 29396559 10.1038/s41598-018-20196-5 PMC5797222

[bib74] Matthews JL , Raina J, Kahlke T, Seymour JR, van Oppen MJH, Suggett DJ. 2020. Symbiodiniaceae-bacteria interactions: rethinking metabolite exchange in reef-building corals as multi-partner metabolic networks. Environ Microbiol. 22:1675. 31943674 10.1111/1462-2920.14918

[bib75] McDevitt-Irwin JM , Baum JK, Garren M, Vega Thurber RL. 2017. Responses of coral-associated bacterial communities to local and global stressors. Front Mar Sci. 4:262.

[bib76] Messyasz A , Rosales SM, Mueller RS, Sawyer T, Correa AMS, Thurber AR, Thurber RV. 2020. Coral Bleaching Phenotypes Associated With Differential Abundances of Nucleocytoplasmic Large DNA Viruses. Front Mar Sci. 7:789.

[bib77] Moore RB , Oborník M, Janouškovec J, Chrudimský T, Vancová M, Green DH, Wright SW, Davies NW, Bolch CJS, Heimann K, et al. 2008. A photosynthetic alveolate closely related to apicomplexan parasites. Nature. 451:959–63. 18288187 10.1038/nature06635

[bib78] Morrow KM , Muller E, Lesser MP. 2018. How Does the Coral Microbiome Cause, Respond to, or Modulate the Bleaching Process?. In: van Oppen MJH, Lough JMeditors. Coral Bleaching. Springer. p.153–88.

[bib79] Motone K , Takagi T, Aburaya S, Miura N, Aoki W, Ueda M. 2020. A Zeaxanthin-producing bacterium isolated from the algal phycosphere protects coral endosymbionts from environmental stress. MBio. 11:e01019–19. 31964724 10.1128/mBio.01019-19 PMC6974559

[bib80] Muscatine L. 1967. Glycerol excretion by symbiotic algae from corals and tridacna and its control by the host. Science. 156:516–9. 17730744 10.1126/science.156.3774.516

[bib81] Nguyen-Kim H , Bettarel Y, Bouvier T, Bouvier C, Doan-Nhu H, Nguyen-Ngoc L, Nguyen-Thanh T, Tran-Quang H, Brune J. 2015. Coral mucus is a hot spot for viral infections. Appl Environ Microbiol. 81:5773–83. 26092456 10.1128/AEM.00542-15 PMC4551247

[bib82] Nitschke MR , Fidalgo C, Simões J, Brandão C, Alves A, Serôdio J, Frommlet JC. 2020. Symbiolite formation: a powerful in vitro model to untangle the role of bacterial communities in the photosynthesis-induced formation of microbialites. ISME J. 14:1533–46. 32203119 10.1038/s41396-020-0629-z PMC7242451

[bib83] O'Brien PA , Andreakis N, Tan S, Miller DJ, Webster NS, Zhang G, Bourne DG. 2021. Testing cophylogeny between coral reef invertebrates and their bacterial and archaeal symbionts. Mol Ecol. 30:3768. 34060182 10.1111/mec.16006

[bib84] Oborník M , Modrý D, Lukeš M, Černotíková-Stříbrná E, Cihlář J, Tesařová M, Kotabová E, Vancová M, Prášil O, Lukeš J. 2012. Morphology, ultrastructure and life cycle of Vitrella brassicaformis n. sp., n. gen., a novel chromerid from the Great Barrier Reef. Protist. 163:306–23. 22055836 10.1016/j.protis.2011.09.001

[bib85] Peixoto RS , Sweet M, Villela HDM, Cardoso P, Thomas T, Voolstra CR, Høj L, Bourne DG. 2021. Coral Probiotics: Premise, Promise, Prospects. Annu Rev Anim Biosci. 16:265. 10.1146/annurev-animal-090120-115444 33321044

[bib86] Pernice M , Raina JB, Rädecker N, Cárdenas A, Pogoreutz C, Voolstra CR. 2020. Down to the bone: the role of overlooked endolithic microbiomes in reef coral health. ISME J. 14:325. 31690886 10.1038/s41396-019-0548-z PMC6976677

[bib87] Rädecker N , Pogoreutz C, Gegner HM, Cárdenas A, Perna G, Geißler L, Roth F, Bougoure J, Guagliardo P, Struck U, et al. 2021a. Heat stress reduces the contribution of diazotrophs to coral holobiont nitrogen cycling. ISME J. 1–9. 10.1038/s41396-021-01158-8 PMC8941099 34857934

[bib88] Rädecker N , Pogoreutz C, Gegner HM, Cárdenas A, Roth F, Bougoure J, Guagliardo P, Wild C, Pernice M, Raina JB, et al. 2021b. Heat stress destabilizes symbiotic nutrient cycling in corals. Proc Natl Acad Sci. 118:e2022653118. 33500354 10.1073/pnas.2022653118 PMC7865147

[bib89] Rädecker N , Pogoreutz C, Voolstra CR, Wiedenmann J, Wild C. 2015. Nitrogen cycling in corals: the key to understanding holobiont functioning?. Trends Microbiol. 23:490–7. 25868684 10.1016/j.tim.2015.03.008

[bib90] Ricci F , Rossetto Marcelino V, Blackall LL, Kühl M, Medina M, Verbruggen H. 2019. Beneath the surface: community assembly and functions of the coral skeleton microbiome. Microbiome. 7:159. 31831078 10.1186/s40168-019-0762-y PMC6909473

[bib91] Robbins SJ , Singleton CM, Chan CX, Messer LF, Geers AU, Ying H, Baker A, Bell SC, Morrow KM, Ragan MA, et al. 2019. A genomic view of the reef-building coral Porites lutea and its microbial symbionts. Nat Microbiol. 4:2090–100. 31548681 10.1038/s41564-019-0532-4

[bib92] Ros M , Suggett DJ, Edmondson J, Haydon T, Hughes DJ, Kim M, Guagliardo P, Bougoure J, Pernice M, Raina J-B, et al. 2021. Symbiont shuffling across environmental gradients aligns with changes in carbon uptake and translocation in the reef-building coral Pocillopora acuta. Coral Reefs. 40:595–607.

[bib93] Rosental B , Kozhekbaeva Z, Fernhoff N, Tsai JM, Traylor-Knowles N. 2017. Coral cell separation and isolation by fluorescence-activated cell sorting (FACS). BMC Cell Biol. 18:30. 28851289 10.1186/s12860-017-0146-8 PMC5575905

[bib94] Sangsawang L , Casareto BE, Ohba H, Vu HM, Meekaew A, Suzuki T, Yeemin T, Suzuki Y. 2017. 13C and15N assimilation and organic matter translocation by the endolithic community in the massive coral porites lutea. R Soc Open Sci. 4:171201. 29308251 10.1098/rsos.171201 PMC5750018

[bib95] Santoro EP , Borges RM, Espinoza JL, Freire M, Messias CSMA, Villela HDM, Pereira LM, Vilela CLS, Rosado JG, Cardoso PM, et al. 2021. Coral microbiome manipulation elicits metabolic and genetic restructuring to mitigate heat stress and evade mortality. Sci Adv. 7:eabg3088. 34389536 10.1126/sciadv.abg3088 PMC8363143

[bib96] Santos HF , Carmo FL, Duarte G, Dini-Andreote F, Castro CB, Rosado AS, van Elsas JD, Peixoto RS. 2014. Climate change affects key nitrogen-fixing bacterial populations on coral reefs. ISME J. 8:2272–9. 24830827 10.1038/ismej.2014.70 PMC4992079

[bib97] Savary R , Barshis DJ, Voolstra CR, Cárdenas A, Evensen NR, Banc-Prandi G, Fine M, Meibom A. 2021. Fast and pervasive transcriptomic resilience and acclimation of extremely heat-tolerant coral holobionts from the northern Red Sea. Proc Natl Acad Sci. 118:e2023298118. 33941698 10.1073/pnas.2023298118 PMC8126839

[bib98] Scharfenstein HJ , Chan WY, Buerger P, Humphrey C, van Oppen MJH. 2022. Evidence for de novo acquisition of microalgal symbionts by bleached adult corals. ISME J. 1–4. 10.1038/s41396-022-01203-0 PMC9122906 35132118

[bib99] Siboni N , Ben-Dov E, Sivan A, Kushmaro A. 2008. Global distribution and diversity of coral-associated Archaea and their possible role in the coral holobiont nitrogen cycle. Environ Microbiol. 10:2979–90. 18707612 10.1111/j.1462-2920.2008.01718.x

[bib100] Siboni N , Ben-Dov E, Sivan A, Kushmaro A. 2012. Geographic Specific Coral-Associated Ammonia-Oxidizing Archaea in the Northern Gulf of Eilat (Red Sea). Microb Ecol. 64:18–24. 22286497 10.1007/s00248-011-0006-6

[bib101] Silverstein R , Cunning R, Baker AC. 2015. Change in algal symbiont communities after bleaching, not prior heat exposure, increases heat tolerance of reef corals. Global Change Biol. 21:236–49. 10.1111/gcb.12706 25099991

[bib102] Smith TP , Thomas TJH, García-Carreras B, Sal S, Yvon-Durocher G, Bell T, Pawar S. 2019. Community-level respiration of prokaryotic microbes may rise with global warming. Nat Commun. 10:5124. 31719536 10.1038/s41467-019-13109-1 PMC6851113

[bib103] Soffer N , Brandt ME, Correa AM, Smith TB, Thurber RV. 2013. Potential role of viruses in white plague coral disease. ISME J. 8:271–83. 23949663 10.1038/ismej.2013.137 PMC3906806

[bib104] Stuart-Smith RD , Brown CJ, Ceccarelli DM, Edgar GJ. 2018. Ecosystem restructuring along the Great Barrier Reef following mass coral bleaching. Nature. 560:92–6. 30046108 10.1038/s41586-018-0359-9

[bib105] Suggett DJ , Smith DJ. 2020. Coral bleaching patterns are the outcome of complex biological and environmental networking. Global Change Biol. 26:68–79. 10.1111/gcb.14871 31618499

[bib106] Tignat-Perrier R , van de Water JAJM, Guillemain D, Aurelle D, Allemand D, Ferrier-Pagès C. 2022. The effect of thermal stress on the physiology and bacterial communities of two key Mediterranean gorgonians. Appl Environ Microbiol. 2:aem0234021. 10.1128/aem.02340-21 PMC8939326 35108095

[bib107] Toller WW , Rowan R, Knowlton N. 2002. Genetic evidence for a protozoan (phylum Apicomplexa) associated with corals of the Montastraea annularis species complex. Coral Reefs. 21:143–6.

[bib108] Tribollet A. 2008. The boring microflora in modern coral reef ecosystems: a review of its roles, in Current Developments in Bioerosion. Wisshak M, Tapanila L, eds. Berlin, Heidelberg: Springer. p. 67–94.

[bib109] Van De Water JAJM , Allemand D, Ferrier-Pagès C. 2018. Host-microbe interactions in octocoral holobionts – recent advances and perspectives. Microbiome. 6:64. 29609655 10.1186/s40168-018-0431-6 PMC5880021

[bib110] van Oppen MJH , Blackall LL. 2019. Coral microbiome dynamics, functions and design in a changing world. Nat Rev Microbiol. 17:557. 31263246 10.1038/s41579-019-0223-4

[bib111] Vanwonterghem I , Webster NS. 2020. Coral Reef Microorganisms in a Changing Climate. iScience. 23:100972. 32208346 10.1016/j.isci.2020.100972 PMC7096749

[bib112] Vasseur C , Bougaran G, Garnier M, Hamelin J, Leboulanger C, Le Chevanton M, Mostajir B, Sialve B, Steyer JP, Fouilland E. 2012. Carbon conversion efficiency and population dynamics of a marine algae–bacteria consortium growing on simplified synthetic digestate: First step in a bioprocess coupling algal production and anaerobic digestion. Bioresour Technol. 119:79–87. 22728186 10.1016/j.biortech.2012.05.128

[bib113] Vega Thurber R , Payet JP, Thurber AR, Correa AMS. 2017. Virus–host interactions and their roles in coral reef health and disease. Nat Rev Microbiol. 15:205–16. 28090075 10.1038/nrmicro.2016.176

[bib114] Vega Thurber R , Willner-Hall D, Rodriguez-Mueller B, Desnues C, Edwards RA, Angly F, Dinsdale E, Kelly L, Rohwer F. 2009. Metagenomic analysis of stressed coral holobionts. Environ Microbiol. 11:2148–63. 19397678 10.1111/j.1462-2920.2009.01935.x

[bib115] Vohsen SA , Anderson KE, Gade AM, Gruber-Vodicka HR, Dannenberg RP, Osman EO, Dubilier N, Fisher CR, Baums IB. 2020. Deep-sea corals provide new insight into the ecology, evolution, and the role of plastids in widespread apicomplexan symbionts of anthozoans. Microbiome. 8:34. 32164774 10.1186/s40168-020-00798-w PMC7068898

[bib116] Wang L , Shantz AA, Payet JP, Sharpton TJ, Foster A, Burkepile DE, Thurber RV. 2018. Corals and their microbiomes are differentially affected by exposure to elevated nutrients and a natural thermal anomaly. Front Mar Sci. 5:101.

[bib117] Wegley L , Edwards R, Rodriguez-Brito B, Liu H, Rohwer F. 2007. Metagenomic analysis of the microbial community associated with the coral Porites astreoides. Environ Microbiol. 9:2707–19. 17922755 10.1111/j.1462-2920.2007.01383.x

[bib118] Wegley L , Yu Y, Breitbart M, Casas V, Kline DI, Rohwer F. 2004. Coral-associated Archaea. Mar Ecol Prog Ser. 273:89–96.

[bib119] Weis VM. 2008. Cellular mechanisms of Cnidarian bleaching: stress causes the collapse of symbiosis. J Exp Biol. 211:3059–66. 18805804 10.1242/jeb.009597

[bib120] Wessels W , Sprungala S, Watson SA, Miller DJ, Bourne DG. 2017. The microbiome of the octocoral Lobophytum pauciflorum: minor differences between sexes and resilience to short-term stress. FEMS Microbiol Ecol. 93:13. 10.1093/femsec/fix013 28175253

[bib121] Weynberg KD , Voolstra CR, Neave MJ, Buerger P, van Oppen MJH. 2015. From cholera to corals: Viruses as drivers of virulence in a major coral bacterial pathogen. Sci Rep. 5:1–9. 10.1038/srep17889 PMC4672265 26644037

[bib122] Xie B , Bishop S, Stessman D, Wright D, Spalding MH, Halverson LJ. 2013. Chlamydomonas reinhardtii thermal tolerance enhancement mediated by a mutualistic interaction with vitamin B12-producing bacteria. ISME J. 7:1544–55. 23486253 10.1038/ismej.2013.43 PMC3721113

[bib123] Yamazaki SS , Nakamura T, Yamasaki H.2008. Photoprotective Role of Endolithic Algae Colonized in Coral Skeleton for the Host Photosynthesis. In: Allen J, Gantt E, Golbeck J, Osmond Beditors.Photosynthesis. Energy from the Sun. Springer: Dordrecht. p. 1391–5.

[bib124] Yang SH , Tandon K, Lu CY, Wada N, Shih CJ, Hsiao SSY, Jane W-N, Lee T-C, Yang C-M, Liu C-T, et al. 2019. Metagenomic, phylogenetic, and functional characterization of predominant endolithic green sulfur bacteria in the coral Isopora palifera. Microbiome. 7:3. 30609942 10.1186/s40168-018-0616-z PMC6320609

[bib125] Ziegler M , Grupstra CGB, Barreto MM, Eaton M, BaOmar J, Zubier K, Al-Sofyani A, Turki AJ, Ormond R, Voolstra CR. 2019. Coral bacterial community structure responds to environmental change in a host-specific manner. Nat Commun. 10:3092. 31300639 10.1038/s41467-019-10969-5 PMC6626051

[bib126] Ziegler M , Seneca FO, Yum LK, Palumbi SR, Voolstra CR. 2017. Bacterial community dynamics are linked to patterns of coral heat tolerance. Nat Commun. 8:14213. 28186132 10.1038/ncomms14213 PMC5309854

